# Biomechanical effects of osteoplasty with or without Kirschner wire augmentation on long bone diaphyses undergoing bending stress: implications for percutaneous imaging-guided consolidation in cancer patients

**DOI:** 10.1186/s41747-018-0082-1

**Published:** 2019-01-28

**Authors:** Roberto Luigi Cazzato, Guillaume Koch, Julien Garnon, Nitin Ramamurthy, Jérémie Jégu, Philippe Clavert, Afshin Gangi

**Affiliations:** 1 Department of Interventional Radiology, Nouvel Hôpital Civil (Hôpitaux Universitaires de Strasbourg, HUS), 1, place de l’Hôpital, 67000 Strasbourg, France; 2 Department of Radiology, Norfolk and Norwich University Hospital, Colney Lane, Norwich, NR4 7UY UK; 3 Laboratoire d’Epidémiologie et de Santé Publique - EA3430, Université de Strasbourg, 4, Rue Kirschleger, 67085 Strasbourg, France; 4 Department of Normal Anatomy, Hôpitaux Universitaires de Strasbourg, HUS France, 1, place de l’Hôpital, 67000 Strasbourg, France

**Keywords:** Bone wires, Diaphyses, Fractures (bone), Polymethyl methacrylate

## Abstract

**Background:**

Osteoplasty has been discouraged in long bones. However, despite a substantial lack of pre-clinical biomechanical tests, multiple clinical studies have implemented a wide range of techniques to optimise long bone osteoplasty. The aim of the present study is to evaluate the biomechanical properties of osteoplasty alone and in combination with Kirschner wires (K-wires) in a cadaveric human diaphyseal model undergoing 3-point bending stress.

**Methods:**

Thirty unpaired human cadaveric hemi-tibia specimens were randomly assigned to receive no consolidation (group 1, *n* = 10), osteoplasty alone (group 2, *n* = 10), or K-wires augmented osteoplasty (group 3, *n* = 10). Specimens were tested on a dedicated servo-hydraulic machine using a 3-point bending test. Fracture load was calculated for each specimen; two-sample Wilcoxon rank-sum tests were used to assess differences between groups.

**Results:**

Median volume of polymethyl methacrylate injected was 18 mL for group 2 (25th–50th percentile 15–21 mL) and 19 mL for group 3 (25th–50th percentile 17–21). There were no significant differences in fracture load between groups 1 and 2 (*z* = − 0.793; *p* = 0.430), between groups 1 and 3 (*z* = − 0.944; *p* = 0.347), and between groups 2 and 3 (*z* = − 0.454; *p* = 0.650). Fractures through the cement occurred in 4 of 30 cases (13.3%); there were no K-wires fractures.

**Conclusions:**

Osteoplasty with or without K-wires augmentation does not improve the resistance of diaphyseal bone to bending stresses.

## Key points

- Osteoplasty is discouraged for long bone tumours due to the risk of secondary fractures.
- Multiple clinical studies have implemented a wide range of techniques to optimise long bone osteoplasty without pre-clinical biomechanical assessment.
- The biomechanical properties of osteoplasty with or without Kirschner wires augmentation have been tested in long bones.
- Osteoplasty with or without Kirschner wires augmentation did not improve the biomechanical properties of long bones.

## Background

Bone-seeking tumours (lung, breast, renal, prostate, and multiple myeloma) account for 45% of commonly diagnosed cancers [1]. Metastatic tumours typically occur in the spine, pelvis, and proximal femur and are complicated by pathological fracture in 17 to 43% of patients [2]. Pathological/impending fractures in long bones are particularly associated with advanced disease [3, 4], severe pain, functional loss, and devastating consequences on quality of life (QoL) and prognosis [3]. Prompt stabilisation is essential to optimise the outcome.

Surgical fixation provides superior construct durability, but is frequently unsuitable for frail oncologic patients, and may negatively impact QoL and survival [5]. Recently, the development of percutaneous imaging-guided therapies including osteoplasty and screw fixation has provided a minimally invasive, effective, and safe alternative to surgery in cancer patients. However, despite excellent palliative results following the consolidation of long bone tumours, osteoplasty alone is associated with frequent secondary long bone fractures, occurring in approximately 8% of cases [6]. This has been attributed to suboptimal material properties of polymethyl methacrylate (PMMA) cement, weaker under bending stress (64 MPa) than under compression (93 MPa); and osteoplasty alone has been discouraged for long bone tumours [7, 8]. Multiple clinical studies have recently implemented a wide range of adjunctive techniques to optimise long bone consolidation [9–18] (Table 1). However, pre-clinical biomechanical studies are substantially lacking.

**Table 1:** Published studies reporting on different consolidative techniques in long bones

First author [reference]	Journal	Year	Number of patients	Target bone	Type of intervention	Secondary fractures (%)
Cazzato [8]	Eur Radiol	2014	51	Long bones	Osteoplasty	9.1
Premat [9]	Eur Radiol	2017	18	Proximal femur	Spindles + osteoplasty	0
Kelekis [10]	CVIR	2016	12	Long bones	25–50 stainless steel micro-needles + osteoplasty	0
Liu [11]	Eur Radiol	2016	36	Long bones	Osteoplasty (19 patients)	26.3
					Osteoplasty + cement-filled catheter in the medullary canal (17 patients)	0
Tian [12]	CVIR	2014	40	Proximal femur	Osteoplasty (19 patients)	23.8
					Osteoplasty + internal fixation with bone trocars stylets (21 patients)	0
He [13]	JVIR	2014	6	Proximal femur	Osteoplasty + internal fixation (bone trocars stylets)	0
Cazzato [14]	Eur J Radiol	2017	11	Proximal femur	Osteosynthesis	0
Lin [15]	Surg Oncol	2015	12	Proximal femur	Osteosynthesis with modified hollow-perforated screws and osteoplasty	8.3
Cornelis [16]	J Orthop Surg Res	2017	10	Proximal femur	Y-STRUT® device	10
Kim [17]	Surg Oncol	2011	15	Humerus	Ender nail fixation and osteoplasty	NR
Kim [18]	Surg Oncol	2014	15	Femur and tibia	Flexible nailing and osteoplasty	NR

The purpose of this study was to evaluate the biomechanical properties of osteoplasty alone and in combination with Kirschner wires (K-wires) in a cadaveric human diaphyseal model subjected to 3-point bending stress as well as to discuss implications for percutaneous imaging-guided consolidation of long bone metastases.

## Methods

Specimens were harvested in compliance with institutional safety regulations. Institutional review board approval was obtained for this cadaveric study in the setting of routine research activity on human cadavers performed at the University Hospital of Strasbourg.

### Bone specimens and study sample

Ten pairs of embalmed human cadaveric tibias were obtained from five cadavers (three male, two female, mean age 75 years, range 67–93 years; mean height 1.67 m, range 1.52–1.78 m) donated to the institutional Anatomy Department of the University Hospital of Strasbourg and preserved by injection of formalin and alcohol solution into the femoral artery. There were no cases of previous surgery or anatomic alteration to the lower legs. Specimens were stored in 20% alcohol solution prior to application and kept moist throughout the study using intermittent saline irrigation.

Each tibia was measured in long axis and axially transected in the mid-diaphysis using an oscillating bone saw. A total of 40 experimental specimens (proximal/distal, right/left hemi-tibias) were obtained. Ten randomly selected specimens were utilised for initial feasibility assessment and excluded from the final study sample. Six were used to assess the technical feasibility of osteoplasty and K-wires augmented osteoplasty procedures and to optimise biomechanical loading protocols. Four specimens were used to assess the technical feasibility of osteoplasty with PMMA-filled catheter augmentation, a novel procedure in which PMMA-filled biliary catheters are used as intramedullary nails to optimise the consolidative properties of PMMA osteoplasty [11]. The technique utilises accessible and familiar equipment and may provide clinical benefits [11]. Unfortunately, it was not possible to replicate this construct in our cadaveric specimens. Despite numerous attempts to manually inject PMMA (using 3-mL syringes at room temperature of 22 °C) into 8–14 Fr biliary catheters positioned longitudinally within the bone specimens using a vertebroplasty trocar (*n* = 4) and extra-osseously (*n* = 1), it was not possible to obtain homogeneous intraluminal cement distribution without causing catheter fracture. This method was therefore not investigated.

The final study sample comprised 30 specimens. Computed tomography (CT) was performed (90 mAs; 120 kVp) to exclude pre-existing focal disease and estimate cortical bone density using x-ray attenuation values (mean of three 1-mm^2^ regions of interest placed in epiphyseal, metaphyseal, and diaphyseal cortex on axial 1-mm slices). Formal bone mineral density estimation and geometric analysis were precluded by the unavailability of peripheral quantitative CT or dual-energy x-ray absorptiometry.

**Fig. 1:**
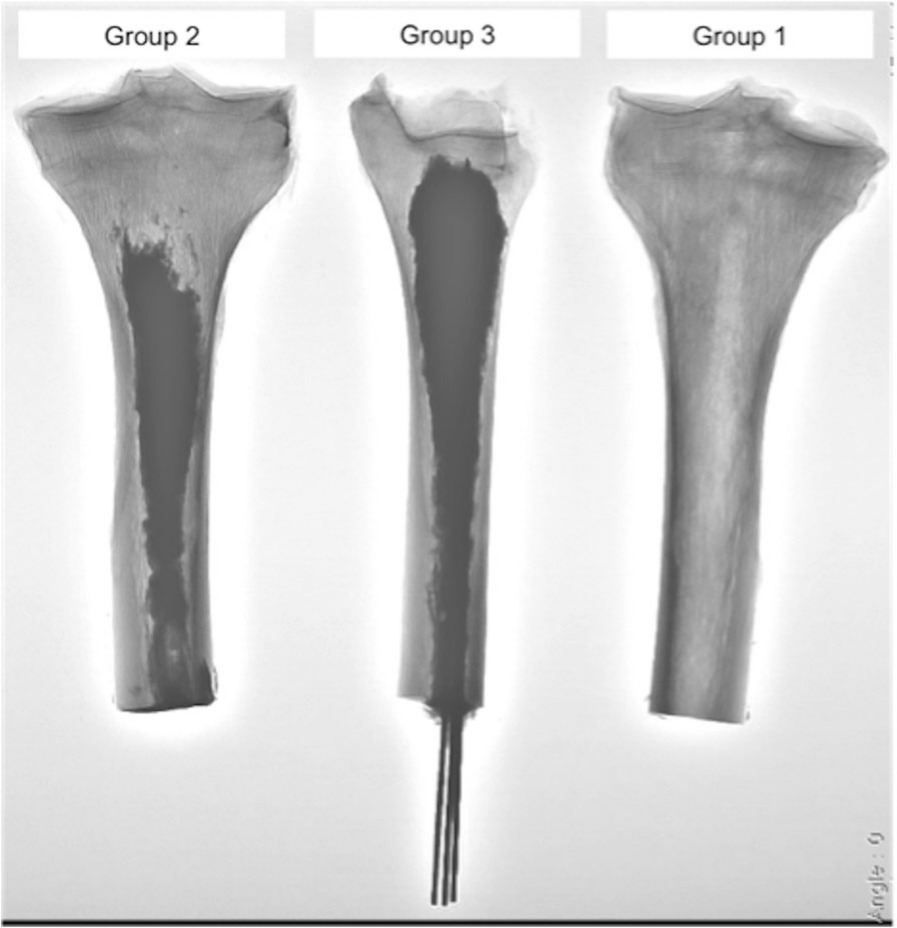
Fluoroscopic image illustrating a bone sample from each experimental group

Bone specimens were randomly assigned to three groups: no consolidative therapy (group1), osteoplasty alone (group 2), and K-wires augmented osteoplasty (group 3) (Fig. 1). Intra-individual controlled pair matching could not be performed due to the paucity of specimens.

### Osteoplasty and K-wires augmentation

Two interventional radiologists with 3- and 5-year experience in bone consolidation performed bone augmentation (groups 2 and 3). For group 2, osteoplasty was performed using a 10-G vertebroplasty needle (Gangi Special Vertebroplasty Needle Set, Optimed, Ettlingen, Germany) advanced within the central long axis of the specimen via the transected surface. Under continuous fluoroscopy, PMMA cement (Osteopal V, Heraeus medical, Wehrheim, Germany) was manually injected using 5-mL syringes until leakage occurred or no further cement could be injected. For group 3, three K-wires were sequentially advanced through the cut bone surface using an electric driver and positioned paramedian to the long axis of the diaphysis in a triangular configuration. Osteoplasty was then performed as above.

### Biomechanical testing and fracture assessment

Due to the limited number of specimens available, only 3-point bending tests were conducted on a dedicated servo-hydraulic machine (INSTRON 8500 plus, INSTRON Corporation, High Wycombe, Buckinghamshire, UK). Although the 3-point bending test may be influenced by shearing stresses, the choice to apply such test was justified by the fact that beams undergoing axial load such as long bones of the lower limbs are also subjected to bending in a direction that is perpendicular to that of the applied axial load (i.e., buckling).

**Fig. 2:**
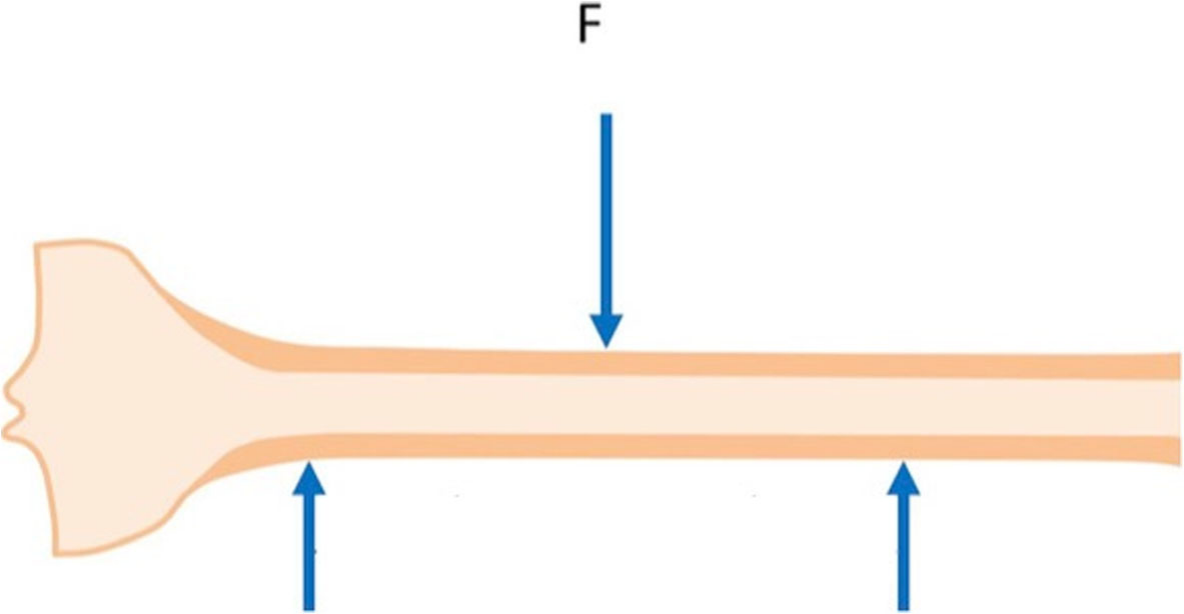
Schematic representation of the 3-point bending test protocol. *F* force

Specimens were positioned horizontally on two fixed supports, lateral aspect facing downwards; the distance between the two points was fixed. A 10-N pre-load was applied midway between the two supports via a metal wedge impact anvil attached to the load cell (Fig. 2). The machine actuator was displaced inferiorly at 50 mm/s until the specimen fractured, while force/displacement data were recorded at 2,500 Hz. The final displacement of the actuator was measured by assuming that the elastic deformation of the machine was negligible compared to that of tested specimens.

Data were transferred to an Excel spreadsheet (Microsoft Corporation, Redmond, WA, USA), force/displacement curves were plotted for each group, and fracture load was automatically calculated by proprietary software. Data regarding Young’s modulus were also provided.

**Fig. 3:**
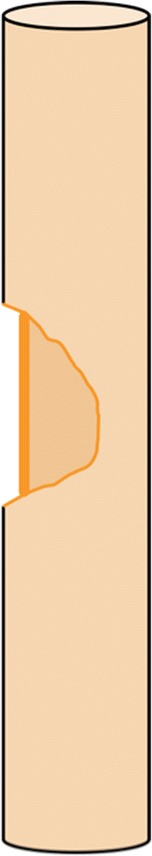
Schematic representation of a *B0* fracture type according to a modified Müller AO classification used in the present study

Fractures involving bone and/or consolidative constructs were documented using two orthogonal plain radiographs. Location and morphology were classified in consensus by two board-certified radiologists (3- and 5-year experience), according to a modified version of the *long bone fracture classification* of the Müller AO Trauma Foundation [19], in which wedge fractures not extending to the opposite cortex were additionally labelled as *B0* (Fig. 3).

### Data collection and statistics

Anatomic origin, length, mean cortical CT attenuation, quantity of PMMA injected, fracture load, Young’s modulus, and type of fracture involving bone/constructs were recorded for each specimen. Results were presented using descriptive statistics, and two-sample Wilcoxon rank-sum test was used to compare fracture load between groups. All statistical analysis was performed with Matlab® (MathWorks, Inc., Natick, MA, USA); *p* values lower than 0.05 were considered significant.

## Results

Specimen anatomic origin, median length, and median estimated cortical CT attenuation value for each group are summarised in Table 2. Median volume of PMMA injected was 18 mL for group 2 (25th–50th percentile 15–21 mL) and 19 mL for group 3 (25th–50th percentile 17–21 mL).

**Table 2:** Baseline characteristics of the experimental specimens

Group	Number of inferior specimens	Number of superior specimens	Number of right specimens	Number of left specimens	Median specimen length (cm, 25th–75th percentile)	Median specimen density (HU, 25th–75th percentile)
1	8	2	6	4	18.375 (17.9–19.25)	1621 (1571–1652)
2	4	6	6	4	18.375 (17.6–19.25)	1600 (1550–1613)
3	5	5	3	7	18.325 (18–19.25)	1606.5 (1550–1632)

*HU* Hounsfield units

**Fig. 4:**
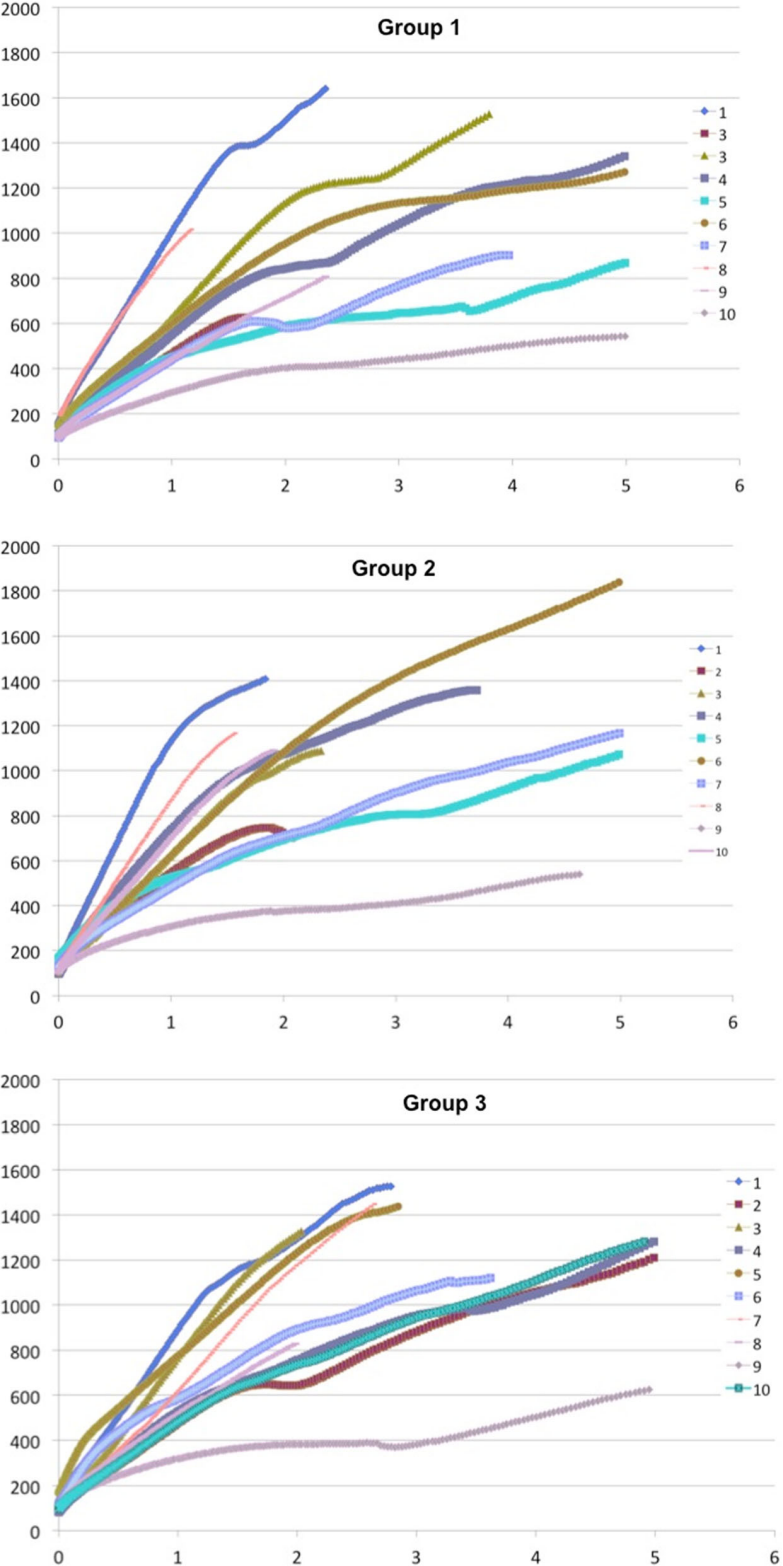
Force/displacement curves. *x-*axis reports specimen displacement (mm); *y-*axis reports the fracture loading force (N). *N* Newton

Force/displacement curves for each group are provided in Fig. 4. Fracture load was numerically higher in consolidated groups than in control specimens (Table 3). However, there was no significant difference between groups 1 and 2 (*z* = − 0.793; *p* = 0.430), between groups 1 and 3 (*z* = − 0.944; *p* = 0.347), and between groups 2 and 3 (*z* = − 0.454; *p* = 0.650).

**Table 3:** Fracture load and Young’s modulus across experimental groups

Group	Fracture load (N)		Young’s modulus (N/m^2^)	
	Mean (SD)	Median (25th–75th percentile)	Mean (SD)	Median (25th–75th percentile)
1	1077.6 (370.16)	1076 (807–1341)	397.15 (140.07)	361.39 (300.65–514.07)
2	1221.5 (338.12)	1166 (1091 – 1391)	444.53 (153.48)	497.88 (309.77–556.35)
3	1230 (292.58)	1280.5 (1119 –1448)	430.73 (140.14)	392.23 (344.59–547.15)

*N* Newton, *SD* standard deviation

The most common type of fracture was the *B0* (14/30, 46.6%) and the *A2* (8/30, 26.6%) (Table 4). Fractures through the cement occurred in 4 of 30 cases (10%), including one from group 2 and three from group 3. There were no K-wires fractures in group 3.

**Table 4:** Type of fractures according to the modified Müller AO Trauma Foundation Long bone Fracture classification (Reference [19])

Group	A0	A1	A2	A3	B0	B1	B2	B3	C0	C1	C2	C3
1	0	2	0	1	6	0	0	1	0	0	0	0
2	0	0	4	1	5	0	0	0	0	0	0	0
3	0	0	4	2	3	0	0	1	0	0	0	0
Total	0	2	8	4	14	0	0	2	0	0	0	0

Young’s modulus did not differ significantly between groups 1 and 2 (*z* = 0.121; *p* = 0.904), between groups 1 and 3 (*z* = 0.338; *p* = 0.728), and between groups 2 and 3 (*z* = 0.148; *p* = 0.881) (Table 3).

## Discussion

Despite the clinical inadequacy of stand-alone osteoplasty for consolidation of long bone pathological/impending fractures [6] and the rapid implementation of numerous alternative techniques [9–18], there has been little evaluation of the biomechanical efficacy of these procedures. In the proximal femur, a few cadaveric studies have shown that femoroplasty effectively consolidates osteoporotic bone subjected to sideways falling [20–22] and may reduce mechanical stress around stance-loaded simulated femoral neck tumours provided that cement filling is adequate [23]. Similarly, the novel Y-STRUT® implant (Hyprevention, Pessac, France; designed to prevent hip fracture) has undergone pre-clinical validation, demonstrating reduced fracture risk during sideways falling [24]. In contrast, there are only three prior animal studies evaluating the biomechanical effects of diaphyseal augmentation. These illustrated a lower bending strength of osteoplasty alone than when combined with bare metal stents or K-wires, in porcine and bovine models with simulated diaphyseal fractures and focal tumours [25–27].

The present study demonstrates that osteoplasty alone or with K-wires augmentation does not confer any consolidative advantage to diaphyseal bone undergoing 3-point bending stress. Fracture load was similar to controls for both composites, consistent with the lack of consolidative and stiffening effects. The stiffness of the tested specimens was slightly increased in composites from group 2 and group 3. Nevertheless, there was no significant difference as compared to controls.

There were four PMMA fractures, consistent with the brittle nature of PMMA and its unsuitability to resist non-compressive loads.

Multiple alternative constructs have been proposed to optimise the consolidation of oncologic long bone tumours, particularly in the proximal femur, with relatively few series treating diaphyseal tumours (only 6% of cases in a recent systematic review [6]).

In several reports, osteoplasty has been combined with dedicated spindles, modified mandrins, and multiple micro-needle mesh to optimise biomechanical resistance of PMMA to multi-directional stresses (*rebar concept*) [10]. Studies in the proximal femur [9, 10, 12] and long bone diaphyses [10] have illustrated good analgesia and restoration of functional status, with either no secondary fractures [9, 10] or fewer than with osteoplasty alone [12], at 6–16-month follow-up. Our study did not demonstrate any beneficial effect of osteoplasty augmented with K-wires, although this may reflect sample limitations, test protocol, or suboptimal composite material properties. Nevertheless, there remains a lack of biomechanical evidence and long-term follow-up to support these procedures.

Other studies have adapted surgical techniques to improve long bone tumour stabilisation. In the proximal femur, percutaneous screw fixation (simulating the *inverted triangle* configuration of orthopaedic procedures) [14, 15, 17] and placement of the Y-STRUT® device simulating a gamma nail [16] have been implemented to treat pathological/impending fractures in selected non-surgical patients. Early results are encouraging, although secondary fracture rates remain considerable (from 6 to 10%) [13, 16]. In contrast, long bone diaphyseal fixation constructs have been largely improvised using interventional radiology equipment. PMMA-filled catheters [11] have been used to simulate the load-sharing action of intramedullary nails (IMN) and augmented osteoplasty of impending fractures. In the series of Liu et al. [11], this resulted in improved analgesia, functional status, and reduced secondary fractures compared with osteoplasty alone. Unfortunately, we were unable to evaluate and replicate this procedure *in vitro*—possibly due to lower ambient temperature, PMMA viscosity differences, and impedance of PMMA flow by the narrow catheter tip and luminal plugging with trabecular bone rather than tumour. Currently, there is insufficient biomechanical and clinical data to support these techniques.

**Fig. 5:**
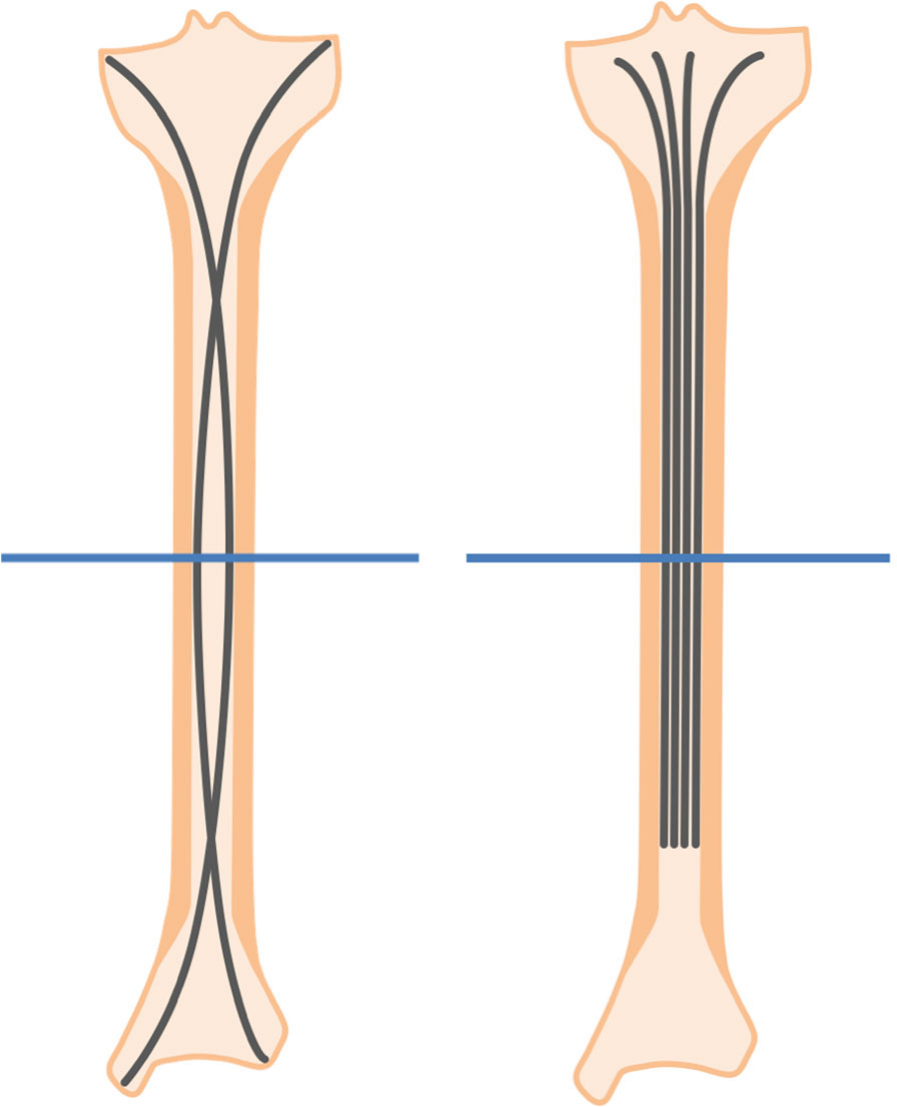
Schematic representation of flexible (left) and bundle (right) intramedullary nailing techniques

The most promising techniques for diaphyseal tumoural consolidation are probably flexible and bundle IMN (Fig. 5); these have been translated from orthopaedic practice to pathological/impending fractures in cancer patients [17, 18] and are already supported by substantial biomechanical and clinical evidence [28, 29]. Flexible IMN produces symmetrical bracing 3-point fixation and achieves (in combination with muscular action) dynamic multi-directional stability. Bundle IMN produces a similar effect via placement of multiple intramedullary pins until the medullary cavity is filled and there is tight compression between the nails and bone [30]. Constructs are typically augmented with PMMA to improve load-sharing and load-bearing properties. Initial clinical results have been promising. Kim et al. [18] performed osteoplasty with flexible IMN in 15 lower limb impending fractures and reported significant palliation, restoration of mobility, and local tumour control (reduction in standardised uptake value at positron emission tomography CT) at 6-month follow-up. Similarly, positive outcomes were also reported for humeral tumours [17]. Disadvantages of the technique include reduced load-sharing properties, lower resistance to torsional/bending stresses [28], and telescoping, compared with standard surgical rigid IMN with proximal/distal interlocking screw fixation [31, 32]. Further studies with long-term follow-up are required to assess these promising interventions.

Our study limitations are mainly related to unavailability of cadaveric specimens. Therefore, neither additional constructs (*e.g.*, flexible, bundle IMN) nor other possible stresses such as torsion or axial load were evaluated. Moreover, our model did not include a bone defect simulating a diaphyseal bone tumour. However, the present study aimed at investigating the biomechanical advantage of osteoplasty or K-wires osteoplasty as compared to the native condition of the target bone. Given the results obtained, one may speculate that similar conclusions might be probably expected also in a model including the same bone defect in all the tested specimens of the three groups. Furthermore, it was not possible to assess bone mineral density and geometric measurements due to unavailability of quantitative CT or dual-energy x-ray absorptiometry or utilise intra-individual controls. However, the majority of fracture risk is accounted for by cross-sectional area of bone rather than mineralisation or morphology [33], and there may be a wide intra-individual heterogeneity even in matched cadaveric samples [34]. The test protocol could have influenced the results in terms of anisotropic effects (varus stress only) and use of hemi-tibias rather than complete bones. However, the choice of 3-point bending test rather than 4-point bending test was made to evaluate the bone strength in the direction perpendicular to the compression axis. As a matter of fact, long bones (especially in the lower limbs) subjected to axial loads can break due to buckling. Finally, 93% of fractures (mainly *A2* and *B0* types) were entirely consistent with a bending mechanism, and spiral *A1* (torsional) pattern was seen in only two cases (7%), suggesting reasonable biomechanical reproducibility.

In conclusion, this study confirms that osteoplasty alone or in combination with K-wires does not improve the resistance of diaphyseal bone subjected to bending stress. Therefore, at the moment, long bone osteoplasty and its variants should still be considered as a suboptimal choice for the consolidation of pathological/impending diaphyseal fractures; as a result, this technique should be avoided in good-prognosis cancer patients and proposed with caution in poor-prognosis, predominantly bed-ridden cancer patients presenting with painful lytic tumours.

## Abbreviations

CT: Computed tomography
IMN: Intramedullary nails
K-wires: Kirschner wires
PMMA: Polymethyl methacrylate
QoL: Quality of life
